# Tumor growth affects the metabonomic phenotypes of multiple mouse non-involved organs in an A549 lung cancer xenograft model

**DOI:** 10.1038/srep28057

**Published:** 2016-06-22

**Authors:** Shan Xu, Yuan Tian, Yili Hu, Nijia Zhang, Sheng Hu, Dandan Song, Zhengshun Wu, Yulan Wang, Yanfang Cui, Huiru Tang

**Affiliations:** 1 Key Laboratory of Pesticide and Chemical Biology, Ministry of Education, College of Chemistry, Central China Normal University, Wuhan 430079, China; 2 CAS Key Laboratory of Magnetic Resonance in Biological Systems, State Key Laboratory of Magnetic Resonance and Atomic and Molecular Physics, National Centre for Magnetic Resonance in Wuhan, Wuhan Institute of Physics and Mathematics, University of Chinese Academy of Sciences, Wuhan, 430071, China; 3 Department of Medical Oncology, Hubei Province Cancer Hospital, Wuhan 430079, China; 4 Collaborative Innovation Center for Diagnosis and Treatment of Infectious Diseases, Zhejiang University, Hangzhou, 310058, China; 5 State Key Laboratory of Genetic Engineering, Collaborative Innovation Center for Genetics and Development, Ministry of Education Key Laboratory of Contemporary Anthropology, Metabonomics and Systems Biology Laboratory, School of Life Sciences, Fudan University, Shanghai, 200438, China

## Abstract

The effects of tumorigenesis and tumor growth on the non-involved organs remain poorly understood although many research efforts have already been made for understanding the metabolic phenotypes of various tumors. To better the situation, we systematically analyzed the metabolic phenotypes of multiple non-involved mouse organ tissues (heart, liver, spleen, lung and kidney) in an A549 lung cancer xenograft model at two different tumor-growth stages using the NMR-based metabonomics approaches. We found that tumor growth caused significant metabonomic changes in multiple non-involved organ tissues involving numerous metabolic pathways, including glycolysis, TCA cycle and metabolisms of amino acids, fatty acids, choline and nucleic acids. Amongst these, the common effects are enhanced glycolysis and nucleoside/nucleotide metabolisms. These findings provided essential biochemistry information about the effects of tumor growth on the non-involved organs.

Cancers collectively remain as a type of most serious diseases globally with high mortality. Even after decades’ efforts, the situation showed no outstanding improvement with more than 14 million newly diagnosed cancer cases and 8 million mortality in 2012 worldwide^1^. In United States alone, for instance, there were 1.6 million new cancer cases in 2015 with half a million cancer-caused deaths^2^. Amongst all cancers, lung cancer accounted for about 13% of the newly diagnosed cancers but 27% of the cancer-caused mortality^2^ with non-small cell lung cancer (NSCLC) accounting for approximately 84% of the lung cancer, half of which are metastatic at time of diagnosis^3^,^4^.

It is now well known that unique metabolic phenotypes are present with largely enhanced glycolysis and lactate elevations in solid tumors, which is known as “Warburg effects”^5^. Choline metabolism involving Kennedy pathway (or cytidine diphosphate choline cycle) is also characteristically different between malignant tumors and non-cancerous tissues with promoted phosphorylcholine biosynthesis in solid malignant tumors due to different demands for cell membrane metabolism^6^. Furthermore, cancer tissues differ considerably from non-cancerous tissues in their metabolism of fatty acid and nucleic acids^7^,^8^. The combined such metabolic alterations have been widely reported in many different cancerous tissues, compared with the adjacent non-involved tissues, including human liver cancer^9^, colorectal cancer^10^,^11^,^12^, lung cancer^13^, thyroid tumor^14^,^15^, bladder cancer^16^, and brain tissues^17^.

It is conceivable that tumorigenesis and tumor growth at a specific organ sites demand more energy and nutrition that can be mobilized from other non-involved organs. Tumor angiogenesis, which generally occurs when tumor reaches about 1–2 mm in diameter, is essential to meet such demands and requirement of other essential supplies including hormones, growth factors and proteolytic enzymes for further growth and metastatic dissemination^18^,^19^,^20^. However, the effects of tumorigenesis and tumor growth on the biochemistry of other non-involved organs remain largely unknown. Such information is conceivably important for understanding not only the effects of tumors on patients as a whole but also the therapeutic effects of effective treatments.

By using the NMR-based metabonomics approaches in this study, we comprehensively analyzed the metabonomic phenotypes of multiple organs (heart, liver, spleen, lung, and kidney) for an A549 lung cancer xenograft mouse model at two tumor growth stages with tumor diameters of about 2 and 10 millimeters. We also analyzed the metabonomic phenotypes of these tumor tissues and the tumor-growth induced concurrent urinary and serum metabonomic changes as complementary information. The objective of this work is to define the effects of lung tumor growth on metabolism of the organs which are not directly involved prior to metastasis.

## Results

### Phenotypes of A549 Xenograft Mice

**Figure 1:**
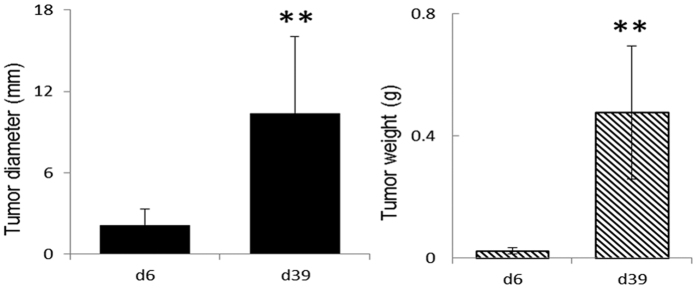
Average tumor sizes (diameter and weight respectively, n = 10) on day-6 (d6) and day-39 (d39) post A549 inoculation. **p < 0.01.

On day 6 post inoculation (d6PI) with A549 cells, tumor size in the A549 xenograft nude mice was about 2 mm in diameter with an average weight of about 0.02 g (n = 10) representing pre-angiogenesis tumor growth. On day 39 post inoculation (d39PI), the tumor sizes (n = 10) were significantly larger than that on d6PI reaching about 10 mm in diameter with an average weight of about 0.48 g (Fig. 1). There were no significant differences in histological features for tissues from heart, liver, spleen, lung and kidney between control and xenograft tumor model mice even on d39PI (Supplementary Fig. S1). This confirmed that on d39PI tumor reached the post-angiogenesis but pre-metastasis growth stage. No significant differences were detected in animal body weights between control and tumor groups (Fig. S2).

### Assignments and Intergroup Differences for Metabolites

The average ^1^H NMR spectra of tissue extracts from ten mouse organs (see Supplementary Fig. S3) showed abundant information of metabolites, which were assigned according to the literature data^21^,^22^,^23^ and confirmed individually with two-dimensional (2D) NMR spectral data^24^ (see Supplementary Table S1). These metabolites included mainly amino acids, lipids (glycerides and cholesterol), ketone body (3-hydroxybutyrate), carbohydrates (glycogen, glucose and mannose), glycolytic metabolites (pyruvate and lactate), TCA cycle intermediates (citrate, succinate and fumarate), bile acids, metabolites of nucleic acids (uridine, uracil, inosine, guanosine, xanthine and hypoxanthine), formate, acetate, taurine, nicotinamide, some acetyl-glycoproteins together with choline metabolites including choline, phosphorylcholine (PC), glycerophosphorylcholine (GPC), dimethylamine, trimethylamine (TMA), dimethylglycine, creatine and ethanolamine (see Supplementary Fig. S3).

**Figure 2:**
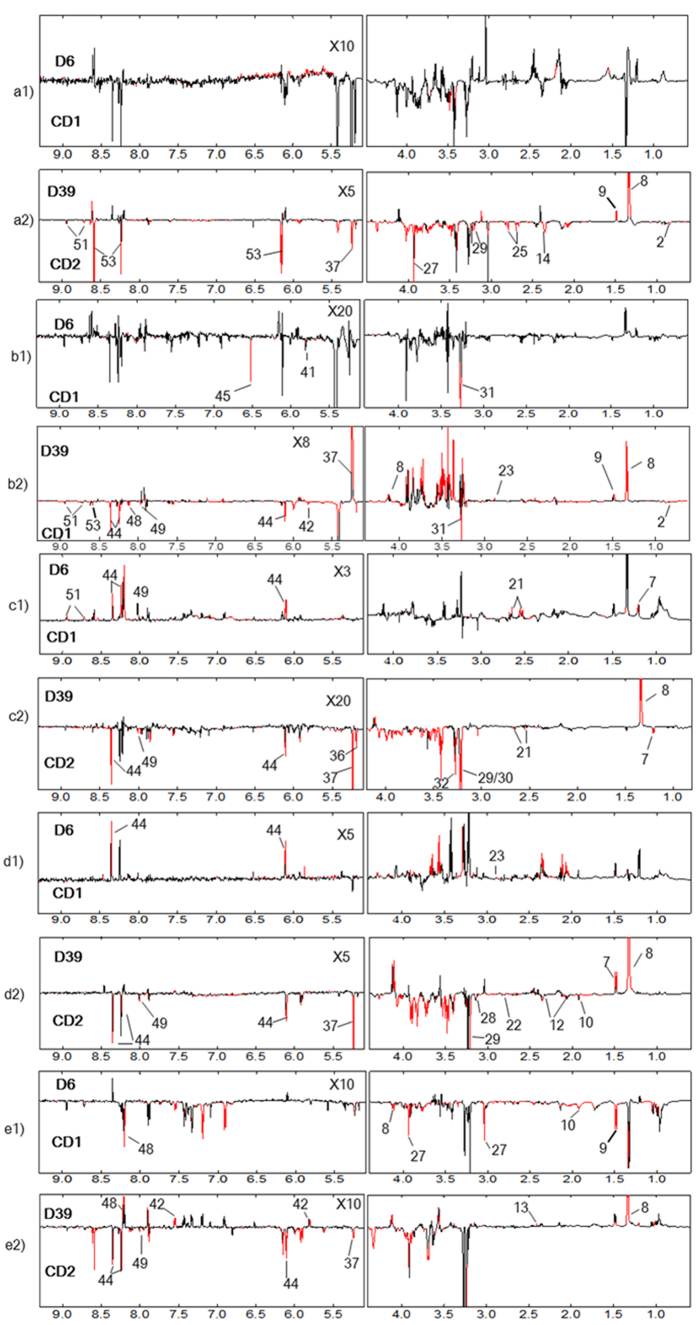
Differential metabograms showing metabonomic differences between the tumor xenograft and control groups in the non-involved mouse organs (n = 10). (A–E) denoted heart, liver, spleen, lung and kidney tissues respectively. D6 and D39 were tumor xenograft groups whereas CD1 and CD2 were their corresponding control groups on day 6 and 39 post A549 inoculations respectively. X3, X5, X8, X10, X20 meant these boxed regions were expanded 3, 5, 8, 10 and 20 times, respectively.

**Figure 3:**
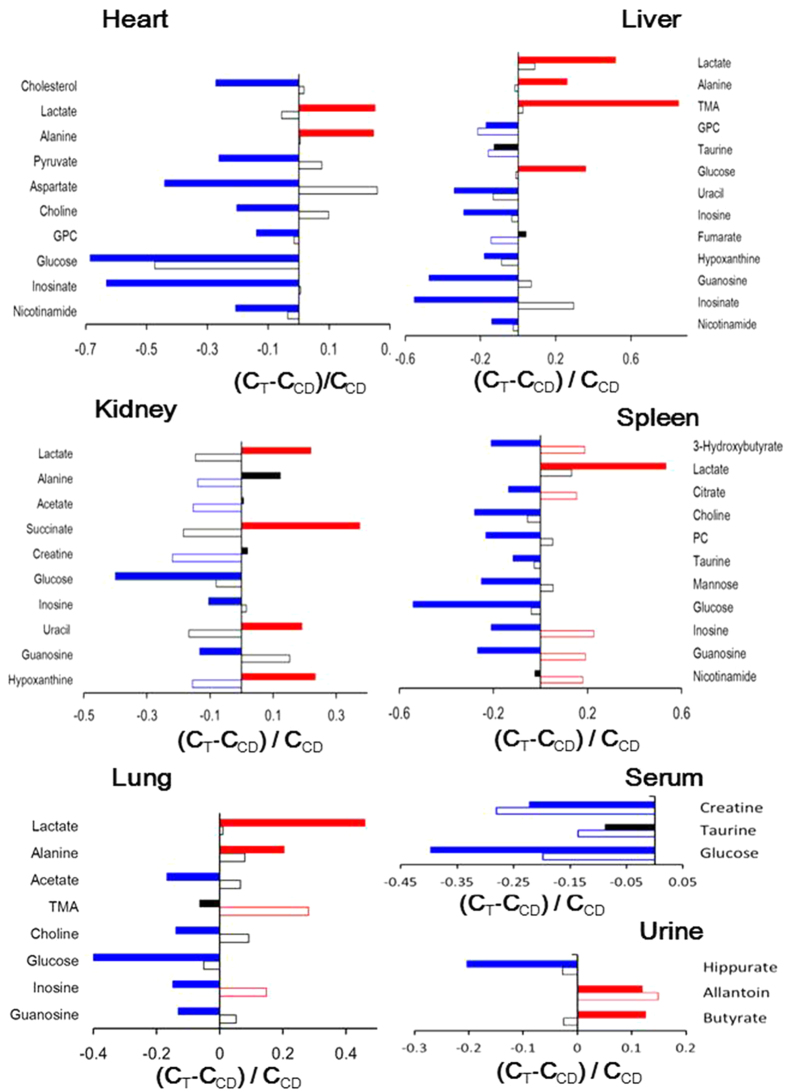
Tumor-growth induced changes of metabolite concentrations in the non-involved organs, serum and urine samples compared to controls (n = 10). C_T_ and C_CD_ denoted metabolite concentration in tumor and control groups, respectively. Empty bars were for tumor and control groups on day 6 post A549 inoculation whereas solid bars for tumor and control groups on day 39 post inoculation. Significant changes were marked with red for increase, blue for decrease and black for no significant changes.

Differential metabograms derived from the multiple univariate data analysis (MUDA)^21^ showed that, on d6PI, only a few metabolites in all these organ tissues had significant differences between the tumor and control groups (n = 10, see Fig. 2). On d39PI, in contrast, many more metabolites showed significant intergroup differences between tumor and control groups (Fig. 2). Signal integrals of the metabolites showing significant intergroup differences (*p* < 0.05) were further analyzed and their corresponding *p-*values were tabulated in Table 1. The tumor-growth induced changes of metabolites were also calculated for both d6PI and d39PI as the ratios of metabolite changes against corresponding control groups (Fig. 3).

### Tumor Growth Induced Metabolic Changes in Heart Tissues

On d6PI, there were no significantly differentiated metabolites between control and the tumor groups. On d39PI, however, lactate and alanine levels were significantly higher whereas the levels of glucose, pyruvate, aspartate, choline, GPC, inosinate and nicotinamide were significantly lower in the tumor group than in controls (Table 1, Fig. 3). Lung tumor growth led to about 25% level increases for lactate and alanine but more than 65% declines for glucose and inosinate. Tumor growth also induced about 20–35% decreases for the levels of aspartate, pyruvate, choline, GPC, and nicotinamide (Table 1, Fig. 3).

### Tumor Growth Induced Metabolic Changes in Liver Tissues

The levels of hepatic GPC and taurine in tumor group showed significant decreases (15–20%) on d6PI compared to control (Table 1, Fig. 3). On d39PI, in contrast, tumor growth caused much more significant metabolic changes highlighted by level elevations for glucose, lactate, alanine and TMA but level declines for guanosine, inosinate, uracil, inosine, hypoxanthine, GPC and nicotinamide. Amongst them, tumor growth caused about 85% level increase hepatic TMA and 55% increase for lactate level with concurrent about 55% decrease for guanosine and inosinate (Fig. 3). The tumor-growth induced level increases for alanine and glucose were about 25–35% whereas level decreases for GPC, uracil, inosine, hypoxanthine and nicotinamide ranged around 15–35% (Fig. 3).

### Tumor Growth Induced Metabolic Changes in Kidney Tissues

On d6PI, tumor growth only caused significant reduction (about 10–20%) in the levels of renal alanine, acetate, creatine and hypoxanthine (Table 1, Fig. 3). In contrast, tumor growth for 39 days induced significant elevations in succinate level (about 35%) together with lactate, uracil and hypoxanthine (20–25%). Tumor-growth for 39 days also caused level reductions for glucose (about 40%) together with inosine and guanosine (about 10–15%) (Table 1, Fig. 3).

### Tumor Growth Induced Metabolic Changes in Spleen Tissues

On d6PI, the levels of 3-hydroxybutyrate, citrate, inosine, guanosine and nicotinamide were about 15–25% higher in spleen tissues of tumor group than in controls (Table 1, Fig. 3). On d39PI, tumor growth caused significant elevation of lactate (about 53%) with concurrent level decline for glucose (about 55%) in spleen. Tumor-growth also resulted in significant decreases (10–30%) in the levels of 3-hydroxybutyrate, citrate, choline, PC, taurine, inosine and guanosine (Table 1, Fig. 3).

### Tumor Growth Induced Metabolic Changes in Non-involved Lung Tissues

The tumor-growth induced metabolic changes were detected in lung tissue even though in this xenograft model tumor only grew at mouse armpit without any detectable histological changes in lung tissue. For example, after A549 inoculation for 6 days, the levels of TMA and inosine in lung tissue of the tumor group were about 28% and 15%, respectively, higher than in controls (Table 1, Fig. 3). In contrast, tumor-growth for 39 days led to significant level elevations for lactate (about 45%) and alanine (about 20%) but decreases for glucose (about 40%) and for acetate, choline, inosine as well as guanosine (about 10–20%) (Table 1, Fig. 3).

### Tumor Growth Induced Metabolic Changes in Urine, Serum and Tumor Tissue Samples

Both urinary and serum metabonomic responses to such tumor growth were mild in both tumor growth stages (Fig. 3). Tumor tissues at two different growth stages showed some significant differences in their choline metabolism (see Supplementary Fig. S4).

## Discussion

Growth of solid tumors often accompanies with induced angiogenesis when tumor size reaches about 1–2 mm in diameter so as to meet the increased demands for energy, nutrition and other essential supplies such as hormones, growth factors and proteolytic enzymes for further growth and metastatic dissemination^18^,^19^,^20^. Such demands inevitably require the mobilization of energy, nutrients and other metabolites from the organs which are not directly involved in tumorigenesis. Therefore, tumor growth at a specific organ site is expected to affect the biochemistry of those non-involved organs as well even before metastasis. So far, little is known about the details of such effects though these effects are potentially important for understanding systems biology of cancer pathogenesis, progression and treatments. Xenograft tumor animal models derived from human cancer cells were shown to be useful for studying the metabonomic phenotypic characteristics of the various corresponding human cancers^25^,^26^.

**Figure 4:**
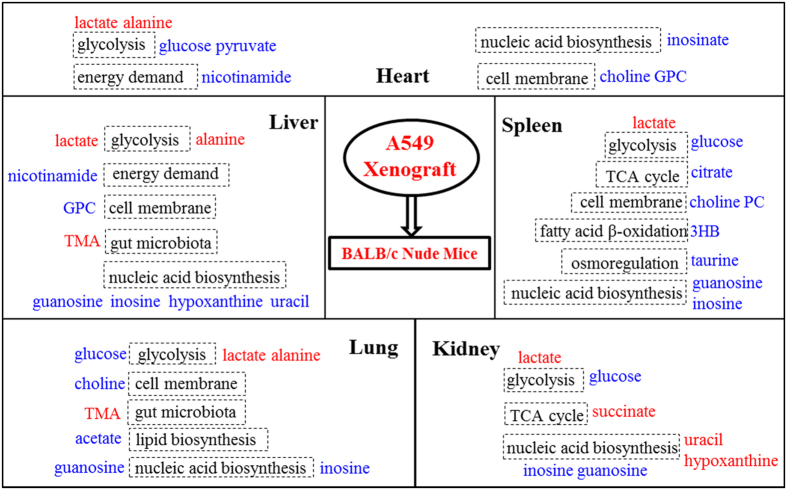
Metabonomic changes in the non-involved organs induced by A549 xenograft tumor growth for 39 days. Significantly elevated and declined metabolites were marked in red and blue, respectively. TMA: Trimethylamine; 3-HB: 3-Hydroxybutyrate; PC, Phosphorylcholine; GPC, Glycerophosphorylcholine.

Here, we employed a classical A549 xenograft tumor mouse model for lung cancer so as to investigate how tumor-growth affects the metabolic phenotypes of the non-involved organs. Such xenograft model was readily established via subcutaneous inoculation of A549 cells into the armpit of nude mice and were commonly used for assessing anti-tumor efficacy of drugs and treatment schedules^27^. We focused on two time points, namely day 6 and 39 post inoculation (d6PI and d39PI). This is because that on d6PI, tumor size was about 2mm in diameter (Fig. 1) representing the stage about angiogenesis whereas on d39PI, tumor size reached about 10 mm in diameter but with no detectable metastasis (see Supplementary Fig. S1) representing more advanced post-angiogenesis but pre-metastatic stages. We found in this work that the growth of xenograft tumor induced substantial metabolic changes in multiple non-involved organs upon angiogenesis (Fig. 4) though such changes appeared to be limited prior to angiogenesis.

Tumor-growth causes significant alterations in glycolysis and TCA cycle in multiple non-involved organs. Tumor growth for 6 days caused limited metabolic changes with kidney and spleen having more metabolic responses than the rest studied organs. Elevations of nicotinamide, citrate and 3-hydroxybutyrate in spleen indicated promotion of fatty acid oxidation with nicotinamide mainly used for nicotinamide adenine di-nucleotide (NAD^+^) biosynthesis^28^,^29^. The decline of creatine in kidney suggested enhancement of energy metabolism with the conversion of phosphocreatine into creatine to produce ATP^30^. Such notion is also supported by observed level declines of serum creatine and glucose for these animals (Fig. 3).

When reached more advanced post angiogenesis stage (i.e., on d39PI), tumor-growth caused lactate elevation in all studied organs indicated the enhanced glycolysis which was supported by the concurrent level decline for glucose. Liver tissue showed elevation of glucose in this case probably due to promoted glycogenolysis as well. The accompanied changes in alanine probably resulted from the same process since pyruvate produced from glycolysis could be converted into alanine by alanine aminotransferase (ALT)^31^ and into lactate by lactate dehydrogenase^32^. This is further supported by significant level declines of serum glucose for the xenograft mice (Fig. 3).

After angiogenesis, tumor growth caused significant changes in nucleic acid metabolism in multiple non-involved organs reflected with significant declines in the levels both purine and pyrimidine metabolites (hypoxanthine, inosine, inosinate, guanosine and uracil) in liver. This probably resulted from the hugely increased demands of nucleotides for biosynthesis of DNA and RNA to ensure cancer cell proliferation during the tumor-growth. Such mobilization of metabolites will partially meet the demands for tumor growth with the rests synthesized by cancer cells themselves via up-regulations of nucleotide biosynthesis using glutamine and glucose as raw materials^33^,^34^,^35^. Such level declines of nucleic acids were less in other organs such as heart, lung and spleen than in liver with it functioning as the major metabolic organ and supplier of metabolites. It is also obvious that tumor-growth effects on nucleic acid metabolism vary with organs. Heart tissue only showed level decline in inosinate (i.e., inosine monophosphate) whereas spleen, lung and kidney had level decreases for inosine and guanosine. The responses of kidney toward xenograft tumor growth (elevation of uracil and hypoxanthine but declines of inosine and guanosine) suggested degradation of nucleosides and oxidative stress for kidney. This is further supported by concurrent elevation of urinary allantoin (Fig. 3).

Moreover, tumor growth induced alterations in choline metabolism in multiple non-involved organs. Choline metabolism is critically important for membrane biosynthesis and degradation through so-called Kennedy cycle^36^. On one hand, choline is converted into PC by choline kinases and further into phosphatidylcholines (PtC) functioning as the major components of cell membrane. On the other hand, GPC derived from PtC (or membrane) degradation can be converted into choline to feed the biosynthesis of PC and PtC again^36^. It is now well known that the PC-to-GPC ratio (PGR) is drastically different for cancer cells (PGR > 1) and normal or benign cells (PGR < 1)^6^. In general, therefore, tumor growth requires large amount of choline and PC to synthesize membrane PtC for rapid cell proliferation^37^ from both the *de novo* biosynthesis of cancer cells and other parts of body. Our observation of consistent level declines for choline, PC and GPC in these non-involved organs during xenograft tumor growth (Table 1, Fig. 3) suggested that demands of membrane biosynthesis in xenograft lung tumor growth (Fig. S4) led to efflux of the choline metabolites from liver, heart and spleen. Currently, it is not clear what affects such efflux-induced losses of metabolites and membrane degradations actually have on the physiological functions of these organs.

It is interesting to observe the tumor-growth caused elevation of TMA in liver and lung tissues. TMA in mammals is largely generated from cholines and carnitines by gut microbiota in gastrointestinal track and then absorbed into liver via enterohepatic circulation. Volatility of TMA also enables it enter lung tissues through so-called air-blood exchange. Therefore, the aforementioned TMA changes in these non-involved organs suggested tumor-growth might also affect the gut microbiota functions as well. This notion is further supported by the tumor-growth induced changes of gut microbial metabolites in our mouse urinary samples such as hippurate and butyrate (Fig. 3). Since urinary hippurate is one of the mammal-microbiota co-metabolites, its changes implies importance of such co-metabolism in tumor growth. The details of such clearly warrant further investigation and the integrative metabonomics and metagenomics approaches^38^,^39^ ought to be feasible, which is currently ongoing.

To sum up, our results indicated that tumor growth had systematic effects on metabolic functions of a number of non-involved organs. Such effects became much more marked during the pre-metastatic post-angiogenesis stage and on more non-involved organs (e.g., liver, spleen, lung, heart and kidney) and probably microbiota functions as well. Xenograft tumor growth promoted glycolysis and membrane degradations, altered TCA cycle, lipid metabolism and nucleotides biosynthesis in these non-involved organs with an outstanding shift of metabolites from the non-involved organs. These findings offered vital information about tumor-growth effects on whole body biochemistry even prior to metastasis which might be of some importance for understanding the tumorigenesis and progression in the systems level.

## Materials and Methods

### Reagents

Dulbecco’s modified Eagle’s medium (DMEM) and trypsin were purchased from Thermo Fisher Scientific Inc. (Beijing, China). Fetal bovine serum (FBS) was obtained from Hangzhou Sijiqing Company. Sodium penicillin and streptomycin were purchased from North China Pharmaceutical Company (Hebei, China). NaH_2_PO_4_ · 2H_2_O, K_2_HPO_4_ · 3H_2_O, K_2_CO_3_ and methanol (all in analytical grade) were purchased from Sinopharm Chemical Reagent Co. Ltd. (Shanghai, China). Deuterium oxide (D_2_O, 99.9%D) and acetyl chloride were obtained from Sigma-Aldrich, Inc. (St. Louis, MO) and sodium 3-(trimethylsilyl) [2,2,3,3-^2^H_4_] propionate (TSP) were obtained from Cambridge Isotope Laboratories, Inc. (Miami, U.S.A.). Phosphate buffer was prepared from NaH_2_PO_4_ and K_2_HPO_4_ with good low-temperature stability^40^ containing 0.001% TSP (w/v) and 80% D_2_O (0.15 M, pH 7.45) and employed as solvent for NMR analysis of tissue extracts.

### Cell Preparation

A549 cells (from human adenocarcinoma alveolar basal epithelial cells) were obtained from China Center for Type Culture Collection in Wuhan University. A549 cells were cultured in a humidified atmosphere with 5% CO_2_ and 95% air at 37 °C in a medium containing DMEM, 10% fetal bovine serum, 100 U/mL sodium penicillin and streptomycin.

### Animal Experiments and Sample Collections

All animal experiments were conducted in accordance with the National Guidelines for Experimental Animal Welfare (the Ministry of Science and Technology, People’s Republic of China, 2006) using a certified SPF facility at the Animal Experiment Center of Hubei Province Cancer Hospital (Wuhan, China). The animal experiment was approved by the local ethic committee of Hubei Province Cancer Hospital. All experimental protocols were in accordance with guidelines of Wuhan Institute of Physics and Mathematics, University of Chinese Academy of Sciences.

Female athymic nude mice (BALB/c, 3–4 weeks old) were obtained from Beijing Vitalriver Experimental Animal Technical Co., LTD. The mice were housed in sterile cages under laminar airflow hoods in a local SPF experimental animal facility with a 12 hr light/dark cycle and constant temperature (to about 25 °C) and relative humidity (to about 50%). All animals were allowed to have free access to normal mouse chow and water. After two weeks acclimatization, mice were inoculated with either A549 cells for the tumor groups or medium for controls as schematically described in Supplementary Fig. S5. For xenograft tumor models, each nude mouse was inoculated with 0.15 mL suspension of A549 cells (5 × 10^7^ cells/mL) subcutaneously into its left armpit. Xenografting mice were randomly divided into two different groups (n = 10), namely, early stage (before angiogenesis) and late stage (post angiogenesis but before metastasis) tumor groups respectively. The former was sacrificed on d6PI whereas the late stage tumor group was completed on d39PI. Two corresponding control groups (n = 10) was treated in the same manner but with 0.15 mL medium. Animal body weights and food intakes were monitored. On d6PI and d39PI, animals were sacrificed with the tumor sizes obtained by weighing tumor tissue and measuring tumor diameters *ex vivo*. These and all other non-involved tissue (heart, liver, spleen, lung and kidney) and biofluid (urine and serum) samples were collected and snap-frozen with liquid nitrogen followed with storage in −80 °C freezer until further analysis. Some tissue samples from two groups (control and tumor group on day 39) were randomly selected and fixed in 10% formalin for histopathological assessments.

### Histopathological Assessment

All tissues from the above selected animals were fixed in formalin followed with staining their micro-sectioned slices with Hematoxylin and Eosin (H&E) methods. All histopathological assessments were conducted by a qualified pathologist microscopically as a paid service.

### Sample Preparations for Metabonomic Analysis

Tissue metabolites were extracted as reported previously^21^,^22^,^23^ with some minor modifications. Briefly, each individual tissue sample (about 50 mg) was weighed and extracted with 600 μL pre-cooled methanol-water mixture (2:1, v/v) using Tissuelyzer II (QIAGEN TissueLyser II, Germany) for 90 s at 20 Hz. The resultant mixture was subjected to ultrasonication for 3 minutes in an ice both followed with centrifugation (11180 × g, 4 °C) for 10 min to collect the supernatant. This extraction procedure was further repeated twice and so-obtained three supernatants from each sample were pooled together followed with another centrifugation. Methanol in the pooled supernatants for all samples was removed *in vacuo* and the resultant extracts were lyophilized. The freeze-dried extract for each sample was individually dissolved in Na-K phosphate buffer (600 μL, pH 7.45) and 550 μL of the supernatant was individually transferred into a 5 mm NMR tube for each sample for further NMR analysis.

### NMR Measurements

All NMR spectra for tissue extracts were acquired at 298 K on a Bruker AVANCE III600 MHz NMR spectrometer equipped with an inverse-detection optimized cryogenic probe (Bruker-Biospin, Germany). One-dimensional ^1^H NMR spectra were acquired for all samples using the first increment of the gradient selected NOESY pulse sequence (RD−90°−t_1_−90°−t_m_−90°−acquisition) with water signal presaturated during both the mixing time (t_m_) of 80 ms and recycle delay (RD) of 2 s. The inter-pulse delay t_1_ was set to about 4 μs and 90° pulse length was adjusted to about 10 μs. 128 transients were recorded into 32k data points with the spectral width of 20 ppm. Serum and urinary spectra were acquired as previously reported^40^,^41^,^42^.

For resonance assignment purposes, a series of 2D NMR spectra were acquired for selected samples and processed as previously reported^28^,^29^. These included ^1^H−^1^H correlation spectroscopy (COSY), ^1^H J-resolved spectroscopy (JRES),^1^H−^1^H total correlation spectroscopy (TOCSY), ^1^H−^13^C heteronuclear single quantum correlation spectroscopy (HSQC), and ^1^H−^13^C heteronuclear multiple bond correlation spectroscopy (HMBC).

### NMR Data Processing and Data Analysis

Free induction decays (FID) were multiplied by an exponential window function with 1 Hz line broadening prior to Fourier transformation. All spectra were phase- and baseline-corrected manually, and referenced internally to the chemical shift of TSP (*δ* 0.00) using Topspin software package (V3.0, Bruker-Biospin, Germany). The *δ* 0.6–9.3 region was bucketed into bins of 0.004 ppm using AMIX software package (V3.9.2, Bruker-Biospin, Germany). The regions of *δ* 4.20–5.20 were discarded prior to analysis to eliminate the effects of imperfect water-saturation. All integrated bins were normalized to the wet weight for tissue samples extracts to represent absolute metabolite quantity in the form of peak-area per mg tissue.

All above normalized bins (i.e., variables) were subjected to either nonparametric tests or the Student’s t-test as appropriate^21^ using an in-house developed MATLAB script. Subsequently, differential metabograms were generated by color-coding all variables (or metabolites) with their corresponding *p*-values in the forms of loadings plots, where the red-colored variables showed significant intergroup differences (*p* < 0.05). Characteristic peaks of the metabolites showing significant intergroup differences were also curve-fitted from original NMR spectra using MestReNova (V8.1.0, Mestrelab Research S. L.) to obtain their peak areas that were subjected to further analysis with the Student’s t-test and Kruskal−Wallis test as appropriate.

The ratios of concentration changes for metabolites showing significant intergroup differences were then calculated as previously reported^22^,^23^ in the form of (C_T_−C_CD_)/C_CD_, where C_T_ and C_CD_ represented the metabolite concentrations in tumor and control groups, respectively. The PC-to-GPC ratio for all samples was individually calculated from the signal integrals of their methyl groups using the same curve-fitting method.

## Additional Information

**How to cite this article**: Xu, S. *et al.* Tumor growth affects the metabonomic phenotypes of multiple mouse non-involved organs in an A549 lung cancer xenograft model. *Sci. Rep.* **6**, 28057; doi: 10.1038/srep28057 (2016).

## Supplementary Material

Supplementary Information

## Figures and Tables

**Table 1:** Significance of the metabolic changes in the non-involved nude mouse tissues induced by the growth of A549 xenograft tumor compared with their corresponding controls (n = 10, with only *p* < 0.05 tabulated).

Metabolites(key)	Heart		Liver		Spleen		Lung		Kidney	
	d6PI	d39PI	d6PI	d39PI	d6PI	d39PI	d6PI	d39PI	d6PI	d39PI
Alanine(9)		0.030		0.000				0.013	−0.038	
Acetate(10)								−0.023	−0.027	
Aspartate(25)		−0.000								
Cholesterol(2)		−0.000								
Choline(29)		−0.000				−0.000		−0.007		
Citrate(21)					0.008	−0.012				
Creatine(27)									−0.005	
Fumarate(45)			−0.034							
Glucose(37)		−0.003		0.009		−0.000		−0.000		−0.015
GPC(31)		−0.009	−0.035	−0.003						
Guanosine(49)				−0.001	0.034	−0.000		−0.006		−0.021
3-HB(7)					0.008	−0.025				
Hypoxanthine(48)				−0.005					−0.021	0.022
Inosinate(53)		−0.000		−0.000						
Inosine(44)				−0.000	0.013	−0.043	0.002	−0.028		−0.013
Lactate(8)		0.001		0.000		0.000		0.001		0.000
Mannose(36)						−0.000				
Nicotinamide(51)		−0.000		−0.006	0.017					
PC(30)						−0.024				
Pyruvate(14)		−0.000								
Succinate(13)										0.020
TMA(23)				0.000			0.013			
Taurine(32)			−0.025			−0.049				
Uracil(42)				−0.000						0.003

*p-*values with negative and no signs meant decreases and increases respectively whilst d6PI and d39PI meant tumor groups on day-6 and -39 post A549 inoculation, respectively. Metabolite keys were marked in Supplementary Fig. S3 When less than 0.001, *p-*values were recorded as 0.000. TMA: Trimethylamine; 3-HB: 3-Hydroxybutyrate; PC, Phosphorylcholine; GPC, Glycerophosphorylcholine.
