# Supplementary material for: Tumor growth affects the metabonomic phenotypes of multiple mouse non-involved organs in an A549 lung cancer xenograft model
Source: Sci Rep. 2016 Jun 22;6:28057. doi: 10.1038/srep28057 (PMC4916411; doi:10.1038/srep28057)
Supplement: Supplementary Information [file srep28057-s1.pdf]

Supplementary data

Tumor growth affects the metabonomic phenotypes of multiple mouse non-involved organs in an A549 lung cancer xenograft model

Shan Xu[†‡#], Yuan Tian[‡#], Yili Hu[‡], Nijia Zhang[†], Sheng Hu[¶], Dandan Song[†], Zhengshun Wu[†], Yulan Wang[‡§], Yanfang Cui[†], Huiru Tang[*Φ]

[†] *Key Laboratory of Pesticide and Chemical Biology, Ministry of Education, College of Chemistry, Central China Normal University, Wuhan 430079, China*

[‡] *CAS Key Laboratory of Magnetic Resonance in Biological Systems, State Key Laboratory of Magnetic Resonance and Atomic and Molecular Physics, National Centre for Magnetic Resonance in Wuhan, Wuhan Institute of Physics and Mathematics, University of Chinese Academy of Sciences, Wuhan, 430071, China*

[Φ] *State Key Laboratory of Genetic Engineering, Collaborative Innovation Center for Genetics and Development, Ministry of Education Key Laboratory of Contemporary Anthropology, Metabonomics and Systems Biology Laboratory, School of Life Sciences, Fudan University, Shanghai, 200438, China.*

[¶] *Department of Medical Oncology, Hubei Province Cancer Hospital, Wuhan 430079, China*

[§] *Collaborative Innovation Center for Diagnosis and Treatment of Infectious Diseases, Zhejiang University, Hangzhou, 310058, China*

# These authors contributed equally to this work

* To whom all correspondence should be addressed.

Prof. Huiru Tang, Tel: +862151630725; E-mail: huiru_tang@fudan.edu.cn

Supplementary Table S1. Metabolites of nude mice in heart, liver, spleen, lung, kidney tissues. [a]s, singlet; brs, broad singlet; d, double; t, triple; m, multiplet; dd, double doublet. [b]H, heart; L, liver; S, spleen; Lu, lung; K, kidney. [c]NAG, N-acetyl-glycoproteins; OAG, O-acetyl-glycoproteins; PC, phosphorylcholine; GPC, glycerophosphorylcholine.

keys	metabolites	moieties	δ^1H (multiplicity)[a]	δ^{13}C	biomatrices[b]
1	Bile acids	CH_3	0.74(brs)	21.1	L, K
2	Cholesterol	CH_3	0.84(s)	21.1	H, L, K
3	Lipids	CH_3	0.90(t)	12.3	H, L, S, Lu, K
		CH_2	1.27(m)	32.6	
		CH_2CH_2CO	1.58(m)	28.1	
		$CH_2C=C$	2.01(m)	29.9	
		CH_2CO	2.23(m)	36.9	
		$C=CCH_2C=C$	2.76(m)	28.3	
		-CH=CH-	5.30(m)	131.5	
4	Isoleucine	δCH_3	0.94(t)	13.8	H, L, S, Lu, K
		βCH_3	1.02(d)	17.5	
		γCH_3	1.48(m)	19.3	
		βCH	1.99(m)	38.8	
		αCH_3	3.79(d)	63.9	
		COOH		177.7	
5	Leucine	δCH_3	0.95(d)	24.3	H, L, S, Lu, K
		$\delta' CH_3$	0.98(d)	25.1	
		γCH	1.69(m)	42.7	
		βCH	1.71(m)	29.5	
		βCH_2	1.72(m)	29.5	
		αCH_3	3.73(dd)	63.6	
		COOH		178.7	
6	Valine	γCH_3	0.99(d)	19.6	H, L, S, Lu, K
		$\gamma' CH_3$	1.04(d)	21.1	
		βCH	2.28(m)	32.3	
		αCH_3	3.61(m)	63.8	
		COOH		184.2	
7	3-Hydroxybutyrate	γCH_3	1.21(d)	24.7	H, L, S, Lu, K
		$\alpha' CH_2$	2.31(dd)	49.6	
		αCH_2	2.42(dd）	49.6	
		βCH	4.16(m)	68.7	
8	Lactate	CH_3	1.33(d)	23.1	H, L, S, Lu, K
		CH	4.12(q)	71.4	
		COOH		185.2	
9	Alanine	CH_3	1.48(d)	19.5	H, L, S, Lu, K
		CH	3.79(q)	53.8	
10	Acetate	CH_3	1.92(s)	26.3	H, L, S, Lu, K

		COOH		177.8	
11	Glutamine	*β*CH2	2.17(m)	29.5	H, L, S, Lu, K
		*γ*CH2	2.54(m)	34.2	
		*α*CH	3.78(m)	57.1	
		COOH		176.8	
12	Glutamate	*β*CH_2	2.07(m)	29.9	H, L, S, Lu, K
		βCH_2	2.12(m)	30.2	
		γCH_2	2.36(m)	36.6	
		*α*CH	3.76(m)	63.1	
		COOH		177.6	
13	Succinate	CH_2	2.41(s)	37.3	H, L, S, Lu, K
		COOH		183.6	
14	Pyruvate	CH_3	2.37(s)	33.6	H, L, S, Lu, K
		COOH		183.1	
15	Malate	CH_2	2.37(dd)	45.8	H, L, Lu, K
		CH_2’	2.68(dd)	45.5	
		CH	4.31(dd)	73.7	
		COOH		183.3	
16	Lysine	*δ*CH_2	1.73(m)	29.5	L, S, Lu, K
		γCH_2	1.48(m)	24.7	
		*β*CH	1.92(m)	32.8	
		εCH_2	3.03(t)	42.4	
		*α*CH	3.77(m)	63.5	
		COOH		178.6	
17	*β*-Mannose	2-CH	3.95(m)	73.5	H, L, S, Lu
		1-CH_3	4.91(d)	96.9	
18	NAG[c]	CH_3	2.04(s)	24.7	L
		C=O		176.2	
19	OAG[c]	CH_3	2.14(s)	17.1	L, K
		C=O		183.8	
20	Methionine	*β*CH_2	2.2(m)	36.5	H, L, S, K
		γCH_2	2.65(t)	31.8	
		*α*CH	3.87(m)	63.7	
21	Citrate	CH_2	2.54(d)	36.1	S, Lu, K
		CH_2'	2.66(d)	36.5	
		COOH		181.7	
22	Dimethylamine	CH_3	2.72(s)	36.1	H, L, S, Lu, K
23	Trimethylamine	CH_3	2.88(s)	47.5	H, L, S, Lu, K
24	Threonine	*β*CH_2	4.26(m)	68.6	L, S, Lu, K
		γCH_3	1.34(d)	25.6	
		*α*CH	3.60(d)	69.5	
		COOH		175.6	
25	Aspartate	*β*CH_2	2.82(dd)	39.6	H, L, S, Lu, K

		$\beta'CH_2$	2.69(dd)	39.6	
		αCH	3.91(dd)	63.6	
		αCOOH		171.9	
26	Dimethylglycine	CH_3	2.93(s)	46.5	H, L, S, Lu, K
		CH_2	3.72(s)	63.6	
27	Creatine	CH_3	3.04(s)	39.9	H, L, S, Lu, K
		CH_2	3.93(s)	56.8	
		C=NH		159.7	
		O=C-N		177.3	
28	Ethanolamine	CH_2NH_2	3.15(t)	44.1	H, L, S, Lu, K
		CH_2OH	3.83(t)	63.6	
29	Choline	$N(CH_3)_3$	3.21(s)	56.9	H, L, S, Lu, K
		OCH2	4.07(m)	58.7	
		NCH_2	3.53(m)	70.5	
30	PC[c]	$N(CH_3)$	3.22(s)	56.9	H, L, S, Lu, K
		N-CH_2	3.60(m)	75.1	
		O-CH_2	4.17(m)	61.2	
31	GPC[c]	N(CH3)	3.234(s)	57.1	H, L, S, Lu, K
		NCH_2	3.69(m)	75.9	
		OCH_2	4.33(m)	62.1	
32	Taurine	CH_2SO_3	3.27(t)	50.6	H, L, S, Lu, K
		CH_2NH_2	3.42(t)	38.3	
33	Glycine	CH_2	3.563(s)	44.5	H, L, S, Lu, K
		COOH		175.1	
34	Triglycerides	OCH	5.19（m)	95.1	L
		OCH_2	4.07(m)	58.6	
		OCH_2'	4.28(m)	88.6	
35	Malonate	CH_2	3.12(s)	56.2	H, L, Lu, K
36	α-Mannose	1-CH	5.19(d)	97.1	H, L, S, Lu
		2-CH	3.94(m)	73.5	
37	α-Glucose	1-CH	5.24（d)	95.2	H, L, S, Lu, K
		2-CH	3.54(dd)	74.7	
		3-CH	3.73(dd)	63.7	
		4-CH	3.40(dd)	72.5	
		5-CH	3.83(m)	74.4	
		6-CH'	3.83(m)	63.7	
38	β–Glucose	1-CH	4.65(d)	99.1	H, L, S, Lu, K
		2-CH	3.25(dd)	77.2	
		3-CH	3.46(m)	79.1	
		4-CH	3.40(dd)	72.6	
		5-CH	3.48(m)	78.9	
		6-CH	3.73(dd)	63.7	
		6-CH'	3.90(dd)	63.7	

39	Tryptophan	9-CH	7.28(t)	132.1	L
		8-CH	7.20(m)	122.7	
		7-CH	7.74(m)	121.1	
		6-CH	7.55(d)	115.1	
40	Glycogen	1-CH	5.42(d）	102.7	L
		4-CH	3.62(m)	75.4	
		2-CH	3.96(m)	72.8	
41	Histidine	C=CH	7.10(d)	120.1	H, L, S, K
		N=CH	7.88(d)	139.1	
42	Uracil	CH	5.81(d)	104.1	H, L, S, Lu, K
		N-CH	7.55(d)	146.1	
43	Uridine	CH2	3.90(dd)	63.9	H, L, S, Lu, K
		N-CH(uracil)	7.89(d)	144.7	
		C-CH(uracil)	5.92(d)	92.3	
		2-H(ribose)	5.91(d)	105.3	
		3-H(ribose)	4.36(dd)	76.7	
		4-H(ribose)	4.24(dd)	68.6	
44	Inosine	CH_2	3.85(dd)	63.9	H, L, S, Lu, K
		4-H(ribose)	4.28(dd)	88.5	
		3-H(ribose)	4.45(dd)	73.4	
		2-H(ribose)	4.78(t)	77.2	
		1-H(ribose)	6.10(d)	91.2	
		2-CH(hypoxanthine)	8.35(s)	143.2	
		8-CH(hypoxanthine)	8.24(s)	149.4	
45	Fumarate	CH	6.52(s)	138.4	H, L, S, Lu, K
		COOH		177.2	
46	Phenylalanine	3OCHN	7.33(dd)	132.7	H, L, S, Lu, K
		2CHCH	7.38(t)	131.6	
		1CHCH	7.43(dd)	133.1	
47	Xanthine	CH	7.90(s)	143.8	H, L, S, Lu, K
48	Hypoxanthine	2-H	8.20(s)	148.6	H, L, S, Lu, K
		8-H	8.21(s)	145.1	
49	Guanosine	CH	8.01(s)	141.1	H, L, S, Lu, K
50	Formate	H-COOH	8.46(s)	174.2	H, L, S, Lu, K
51	Nicotinamide	2-CH	8.94(t)	150.7	H, L, S, Lu, K
		4-CH	8.26(m)	139.7	
		5-CH	7.60(dd)	127.5	
		6-CH	8.72(dd)	154.8	
52	Tyrosine	o-CH	6.90(d)	119.5	L, S, Lu, K
		m-CH	7.19(d)	134.4	
		βCH_2	3.06(dd)	38.4	
		$\beta' CH_2$	3.20(dd)	38.8	
		αCH	3.95(dd)	59.4	
53	Inosinate	CH_2	4.02(dd)	66.3	H, L, S, K

	4-H(ribose)	4.37(dd)	87.6
	3-H(ribose)	4.52(dd)	73.7
	2-H(ribose)	4.78(t)	76.9
	1-H(ribose)	6.15(d)	90.4
	2-CH(hypoxanthine)	8.57(s)	142.8
	8-CH(hypoxanthine)	8.24(s)	149.3

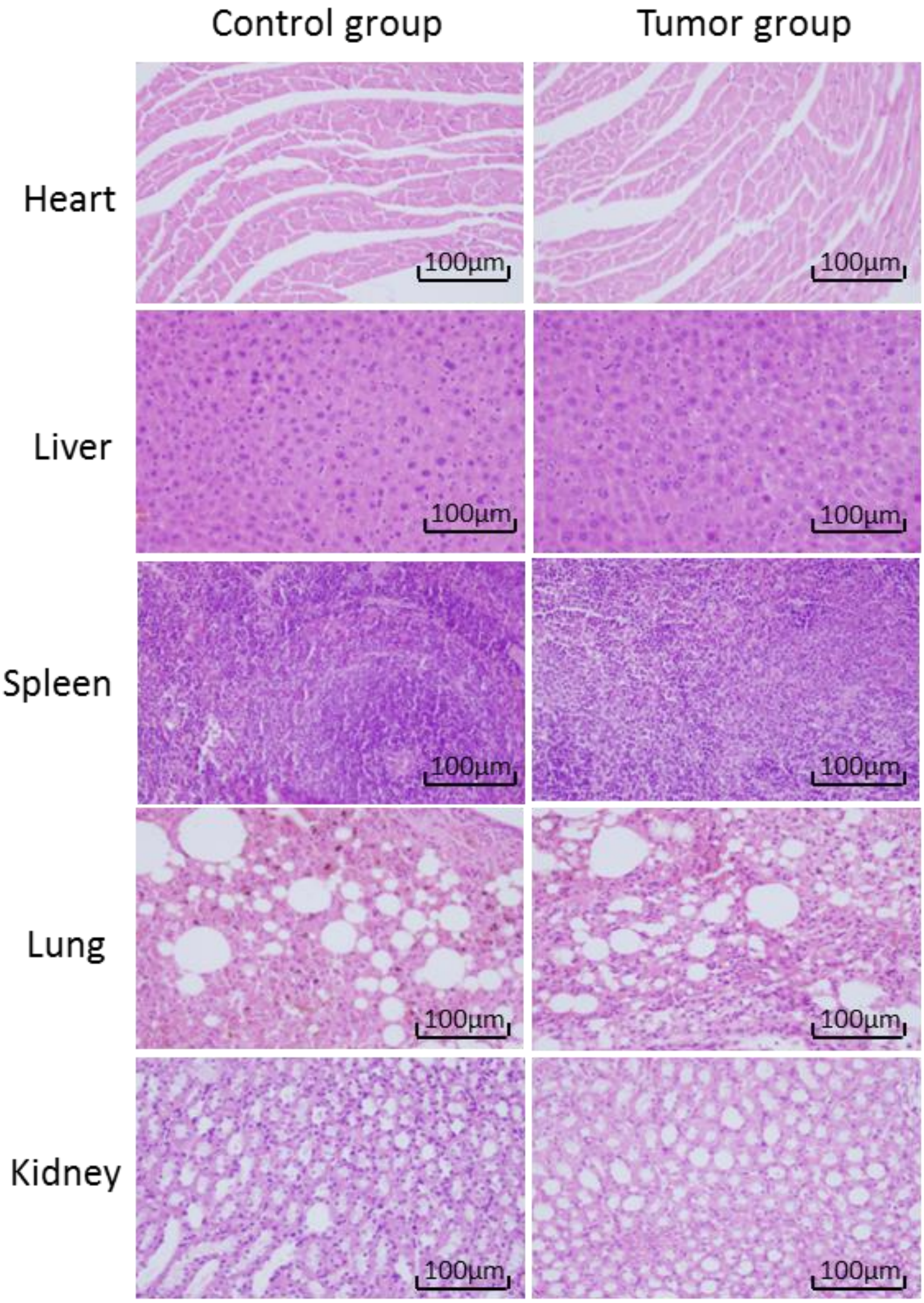

Supplementary Figure S1. Histopathological results for heart, liver, spleen, lung, kidney tissues from the control and tumor groups on day 39 after A549 inoculation.

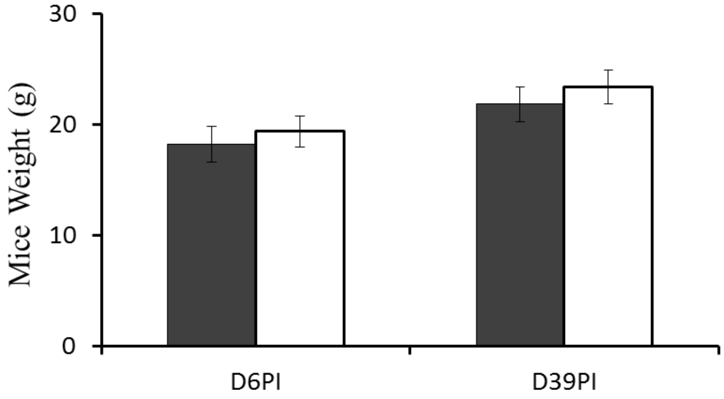

Supplementary Figure S2. Animal body weights for control (solid bars) and tumor groups (empty bars) on day 6 and 39 post A549 inoculations respectively. No significant differences between controls and tumor group at both time points.

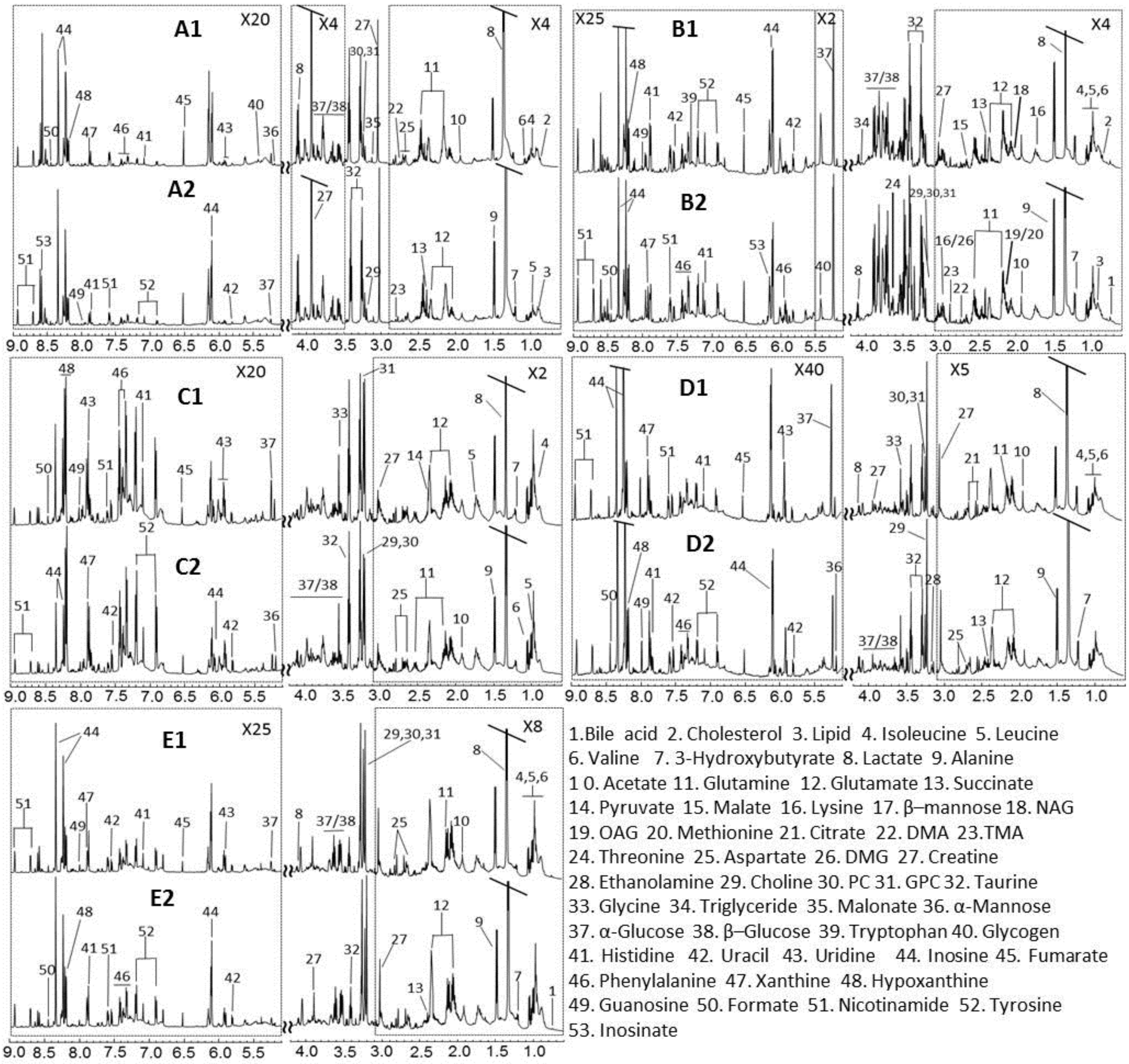

Supplementary Figure S3. Average 600 MHz ^{1}H NMR spectra for the tissue extracts of heart (A), liver (B), spleen (C), lung (D) and kidney (E) from ten animals. A1, B1, C1, D1 and E1 were sampled on day 6 post A549 inoculation whereas A2, B2, C2, D2 and E2 were sampled on day 39 post inoculation. Regions at δ 5.1-5.5 in B1-B2 and δ 0.6-3.1 in C1-C2 were vertically expanded 2 times whereas δ 0.6-3.1, δ 3.5-4.2 in A1-A2 and δ 0.6-3.1 in B1-B2 were expanded 4 times. Regions at δ 0.6-3.1 and δ 5.1-9.0 in D1-D2, δ 0.6-3.1 in E1-E2 were vertically expanded 5, 40 and 8 times, respectively. Regions at δ 5.1-9.0 in A1-A2 and C1-C2 were vertically expanded 20 times whereas δ 5.5-9.0 in B1-B2 and δ 5.1-9.0 in E1-E2 were expanded 25 times. NAG: N-acetyl-glycoproteins; OAG: O-acetyl-glycoproteins; DMA: Dimethylamine; DMG: Dimethylglycine; TMA: Trimethylamine; PC: Phosphorylcholine; GPC: Glycerophosphorylcholine.

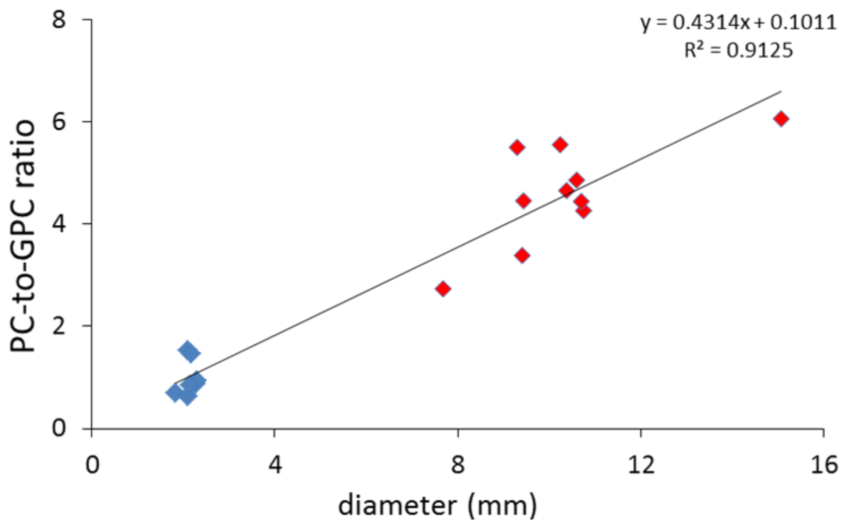

Supplementary Figure S4. The PC-to-GPC ratios for tumor tissues as a function of tumor size (blue: tumor on d6PI; red: tumor on d39PI).

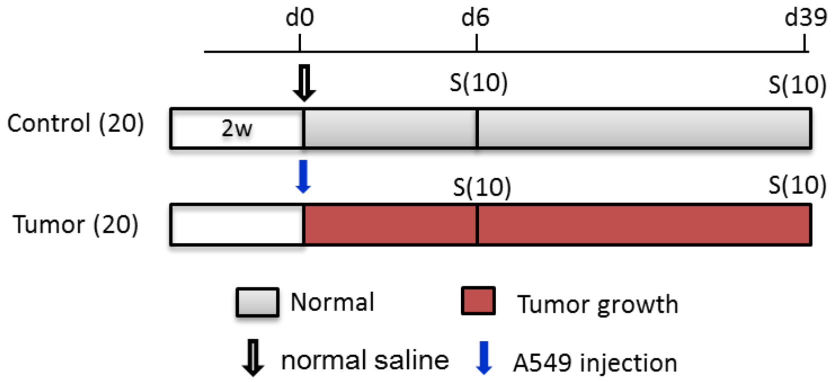

Supplementary Figure S5. Schematic representation of the animal experiments. 2w: 2 weeks; d6, d39: day-6 and day-39; S: sacrifice animal; all number in parentheses: number of animals in that group.
